# Balancing Training and Wellbeing - Psychiatry Trainees’ Views

**DOI:** 10.1192/j.eurpsy.2026.11962

**Published:** 2026-07-31

**Authors:** R. I. Sirisinghe, A. Rios, A. Landers, V. Akpubi

**Affiliations:** Department of Psychiatry, University Hospital Waterford - HSE, Waterford, Ireland

## Abstract

**Introduction:**

Psychiatry training offers rich clinical exposure but also presents challenges that extend beyond knowledge and skills (Karageorge A et al., 2016) Positive training experiences are influenced by high-quality supervision, supported autonomy, and the opportunity to witness patient recovery (Maisonneuve JJ et al., 2014. BMC Med Educ. 14:27., Mendall J et al., 2024. Eye (Lond)). Understanding trainees’ perspectives helps identify strengths in training, informs initiatives to enhance wellbeing, and supports professional development.

**Objectives:**

This cross-sectional study explores psychiatry trainees’ experiences in Ireland regarding confidence, supervision, teaching, autonomy, and work–life balance. The goal is to capture trainee perspectives to inform ongoing development and enhancement of psychiatry training, highlighting factors that contribute to a positive learning experience while maintaining anonymity.

**Methods:**

An anonymous survey was distributed to psychiatry non-consultant hospital doctors (NCHDs). It addressed clinical preparedness, supervision, teaching, autonomy, work–life balance, and wellbeing. Participation was voluntary, responses were fully anonymous, and no identifiable data were collected.

**Results:**

Preliminary findings from a sample of trainees (N < 30) indicate that most feel academically prepared and confident applying theory to practice (72.7% see enough cases; 90.9% confident in clinical application). All respondents (100%) felt training provided adequate exposure to manage patients confidently. Most felt supported by supervisors (81.8%) and found teaching sessions helpful (72.7%), suggesting scope to enhance some teaching. Work–life balance was universally recognised as important, though 54.5% reported maintaining it consistently, while 27.3% did not and 18.2% were unsure. Most trainees indicated that more than two hours daily of restorative activities support wellbeing.

Data collection is ongoing, and additional responses will be incorporated to provide a more comprehensive overview of trainee experiences.

Graphical analyses are pending and will be finalised once additional data have been collected

**Conclusions:**

As these are preliminary results, gathered from a selection of training sites in Ireland, no definitive conclusions are drawn at this stage. Early findings suggest that psychiatry trainees are largely satisfied with clinical exposure, supervision, and teaching. Work–life balance appears to be an area warranting further exploration to better understand trainees’ experiences and support wellbeing. Ensuring anonymity and emphasizing continuous development, this ongoing project aims to provide constructive insights to strengthen trainee confidence, resilience, and professional growth, informing strategies to enhance psychiatry training.

**Disclosure of Interest:**

None Declared

